# Mood and Memory Function in Ovariectomised Rats Exposed to Social Instability Stress

**DOI:** 10.1155/2013/493643

**Published:** 2013-06-13

**Authors:** Badriya Al-Rahbi, Rahimah Zakaria, Zahiruddin Othman, Asma' Hassan, Sangu Muthuraju, Wan Mohd Zahiruddin Wan Mohammad

**Affiliations:** ^1^Department of Physiology, School of Medical Sciences, Universiti Sains Malaysia, 16150 Kubang Kerian, Kelantan, Malaysia; ^2^Department of Psychiatry, School of Medical Sciences, Universiti Sains Malaysia, 16150 Kubang Kerian, Kelantan, Malaysia; ^3^Department of Anatomy, School of Medical Sciences, Universiti Sains Malaysia, 16150 Kubang Kerian, Kelantan, Malaysia; ^4^Department of Neuroscience, School of Medical Sciences, Universiti Sains Malaysia, 16150 Kubang Kerian, Kelantan, Malaysia; ^5^Department of Community Medicine, School of Medical Sciences, Universiti Sains Malaysia, 16150 Kubang Kerian, Kelantan, Malaysia

## Abstract

This study aims to compare the effects of social instability stress on memory and anxiety- and depressive-like behaviour between sham-operated controls and ovariectomised (OVX) rats. Forty adult female Sprague-Dawley rats (8 weeks old) were randomly divided into four groups, (*n* = 10 per group). These were non-stressed sham-operated control rats, stressed sham-operated control rats, non-stressed OVX rats, and stressed OVX rats. The rats were subjected to social instability stress procedure for 15 days. Novel object recognition, open field, and forced swim tests were conducted after the stress procedure. Serum estradiol, ACTH and corticosterone levels were measured using commercially available ELISA kits. Lower serum estradiol level and uterine weight with higher weight gain were observed in OVX rats compared to sham-operated controls. Serum ACTH, and corticosterone levels were higher in stressed compared to non-stressed groups. Memory deficit and anxiety- and depressive-like behaviour were significantly increased in stressed compared to non-stressed OVX rats but these changes were not seen in sham-operated controls. These results suggest that the high circulating corticosterone acts synergistically with low circulating estradiol to exert negative effects on mood and memory function.

## 1. Introduction

Females have a higher prevalence to develop anxiety, depression or cognitive deficit [1–8] compared to males. Additionally, women have a higher probability of developing major depression, and memory deficit when they are 45–55 years old than at other ages [9, 10], suggesting that alteration in the sex hormones levels may signify a risk factor for the development of depressive symptoms [11–13] and cognitive deficit [14, 15].

Ovarian hormones had been shown to exert antidepressive [16, 17] and anxiolytic [18–20] effects and cognitive benefits [21, 22] in various animal studies. Hence, the administration of these hormones to ovariectomised (OVX) rodents results in decreasing depressive-like behaviour as shown by decreasing immobility time in the forced swimming test [23–27], declining anxiety behaviour as shown by increasing time spent in open arms in the elevated plus maze test [17, 19, 20, 28, 29], and improving novel object recognition memory [30].

These results highlight the importance of ovarian hormones in mediating the cognition and anxiety- and depressive-like behaviour, as well as the sensitivity of these behavioural tasks to female hormonal states. Since stressful experiences may also increase the risk of affective disorders [31–36] and cognitive deficit [37], it is important to determine the possible interaction of ovarian hormones and stressful experience. 

Stress is one of the major contributors in increasing individual's vulnerability to affective disorders [38] and memory deficit [39]. Stress-induced changes in adrenal steroid hormones may impair the brain function by acting directly upon neuronal function. Adrenal steroids may also regulate monoamines such as serotonin which are essential elements in the response of the brain to stress. Both monoamines and steroids may alter the expression of peptides in the limbic system, and these peptides may determine specific patterns of response to different stressors [40]. In the majority of studies, stress increases emotionality and impairs cognition in males, whereas in female animals stress alleviates emotionality and enhances cognition, depending on learning task and stressor [41].

Despite serious arguments for using female animals when designing models for stress-related psychiatric disorders and pharmacotherapy, the application of these animal models has contributed substantially to the understanding of the role of social stress in vulnerability to affective disorders and cognitive deficit in female animals. The main argument for using female animals is their estrous cycle, which is characterized by a distinct secretion pattern of estrogens and progesterone [42]. Thus, this study used OVX rats' model to examine the effects of social instability stress on memory and anxiety- and depressive-like behaviour. 

## 2. Materials and Methods

### 2.1. Animal

Forty adult female Sprague-Dawley rats at approximately 8 weeks old, with a body weight of 200 ± 20 g, were obtained from the Laboratory Animal Research Unit, Universiti Sains Malaysia (USM). All rats were housed in polypropylene cages (32 × 24 × 16 cm), exposed to 12 hour light-dark cycles, maintained at a room temperature of 23°C, and had free access to food and water. The experimental protocol was approved by the Research and Ethics Committee, USM. The protocol was approved by the ethics committee (USM/Animal Ethics Approval/2011/(64)(272)) of this university, in accordance with the internationally accepted principles for laboratory animal use and care.

The rats were randomly divided into four groups (*n* = 10 per group) as follows: (i) non-stressed sham-operated control, (ii) stressed sham-operated control, (iii) non-stressed OVX, and (iv) stressed OVX. The rats' body weights were recorded at the start of this study (pre) and at sacrifice (post). The rats were subjected to social instability stress procedure and behavioural tests. They were killed by decapitation upon completion of the behavioural tests. When the rats were killed, ovariectomy was verified by visual inspection, uterine weights were determined, and 10 mL of blood samples was collected immediately from trunk blood. All blood samples were left to clot for 2 hours prior to centrifugation for 15 minutes at 4000 rpm/minutes (EBA 21, Hettich GmbH & Co. KG, Tuttlingen, Germany). Approximately 3 mL of serum was collected and stored at –20°C until assay.

### 2.2. Surgery Procedure

Twenty rats were bilaterally OVX through a dorsal incision under anaesthesia (90 mg/kg ketamine and 5 mg/kg xylazine, intraperitoneally). The other 20 rats were sham-operated; that is, the ovaries were not removed. After the operation, the rats were kept in an individual cage to avoid any interactions which might pose bleeding or poor wound healing for up to 10 days. The groups were reformed and all the rats were left undisturbed for two months as a recovery period.

### 2.3. Social Instability Stress Procedure

The social instability stress procedure was conducted eight weeks after the ovariectomy [43–46]. The stress procedure consisted of alternating isolation and crowding phases for 15 days as previously described [47]. The experiment started and ended with an isolation phase, and each phase lasted for 24 hours. Eight rats (three males and five females) were held per cage during a crowding phase. Rats' behaviour was videotaped for the initial 30 minutes of each crowding phase. Biting attacks, dominant postures and fighting for food were counted [48–50].

### 2.4. Behavioural Tests

All the behaviour tests were performed after the social instability stress procedure. The tests were performed during dark (active) phase in a ventilated and soundproof room that was maintained at a constant temperature (23°C). The lighting intensity was maintained at a constant lux throughout the procedure with two 150 watt lamps.

*(a) Novel Object Recognition (NOR) Test*. This test is normally used to assess cognitive alterations associated with ageing, genetic manipulations, or drug treatments. The chamber was an open field apparatus (60 × 60 × 30 cm). Firstly, all animals were submitted to a habituation session for three days during which they were placed in the empty open field and left to freely explore the field for 10 minutes. During training sessions, two identical objects (A1 and A2) were placed in the field, and the rat was allowed to explore freely for 10 minutes as described in previous studies [51, 52]. Time spent exploring each object was recorded manually. For test sessions, animals were tested for memory retention 2 hours commencing the training session (short-term memory/retention, STM). In the STM test, the rats explored the open field for 5 minutes in the presence of one familiar (A1 or A2) and one novel (B) object. The location of the object was alternated with each new animal; it was approximately placed in 50% of the trials on the right side and 50% on the left side of the field. The same test was repeated 24 hours after the training session and this is known as long-term memory/retention, LTM.

All objects were made of plastic toys and had a height of about 5 cm. Objects presented similar textures, colours, and sizes, but distinctive shapes. The objects were positioned in two adjacent corners, 10 cm from the walls. Between tests, the objects were cleaned with a 10% ethanol solution to mask any olfactory cues.

Exploration was defined by sniffing or touching the object with the nose. Sitting on the object was not considered exploration [53]. Total exploration time(s) of the familiar and novel objects was recorded and used to calculate a discrimination index (time spent with novel object (B) − time spent with familiar object (A))/(total time exploring both objects) for training and test sessions [54]. This index was used to measure recognition memory [55]. The exploration of each object was expressed as the percentage of total exploration time. Increased exploration time of the novel object or better preference to novel objects was interpreted as successful retention of memory for the familiar object. An absence of any variance in the exploration of the two objects was interpreted as memory deficit [56].

*(b) Open Field Test (OFT)*. The OFT arena consisted of a 35 cm high transparent plastic wall and a floor with a surface area of 120 × 120 cm. The floor contained white lines that divided the surface area of the chamber into 16 equal squares [57] and three fluorescent lights provided diffuse overhead illumination. The animals were tested in a quiet room, and rats' activity over a 10 min period was recorded using a digital camcorder placed at a control panel stationed 5 m from the testing apparatus for later behavioural analysis. Between each rat, the apparatus was cleaned with 70% alcohol to eliminate the possible bias due to the odour that could be left by the previous subject.

Recorded video was later scored by an experienced observer who was blind to the condition of the animals and calculated the mean number or duration of the following parameters: (i) movement including rearing events (number of vertical activity), freezing time (seconds), and grooming time (seconds), (ii) locomotor activity, that is, time spent crossing the line (seconds), and (iii) autonomic nervous system responses including defecation (number of fecal boli) and number of face-washing events.

*(c) Forced Swim Test (FST)*. The animals were first trained for 15 minutes in each training session for the duration of two days followed by a test session 24 hours later. All rats were tested in the same brightly lit room. Tests were conducted between 10:00 and 17:00 hours. All rats were individually placed into glass cylinders (40 cm in height, 18 cm in diameter) filled with water (23°C) to a level of 30 cm for 5 minutes. The water level was purposely chosen at a higher level than in the procedure described by Porsolt et al. [58] in order to prevent the rats from supporting themselves by touching the bottom with their hind limbs or tails during the swimming sessions [59]. The animals were forced to swim in the cylinders for 5 minutes during the test session. The three behaviours which were recorded and scored were immobility, swimming, and struggling. These three behaviours are defined as follows: (1) immobility—floating in the water and making those movements which are only necessary to keep their heads above the water, (2) swimming—making active swimming motions, and (3) struggling—rats making active attempts to escape from the cylinder, including visually searching for the escape routes and diving [50]. After completion of the test, the rats were dried gently with a towel and were returned to their home cages.

### 2.5. Estimation of Serum Estradiol, ACTH, and Corticosterone Levels

Serum estradiol, adrenocorticotropic hormone (ACTH), and corticosterone levels were measured using specific ELISA kit (Creative Diagnostics, Shirley, NY, USA) according to the manufacturer's instructions. Briefly, 100 *μ*L of serum sample was added into each well followed by 100 *μ*L of enzyme-labeled estradiol/ACTH/corticosterone. The plate was incubated at 37°C for 90 min. Following incubation, the wells were carefully washed. 100 *μ*L of biotin-antibody working solution was added into each well and then incubated at 37°C for 60 min. Following three washes, 100 *μ*L of horseradish peroxidase (HRP) was added into each well and then incubated at 37°C for 30 min. Next, 100 *μ*L of 3, 3′, 5, 5′-tetramethylbenzidine (TMB) reagent was added into each well and then incubated at room temperature for 20 min, which resulted in the development of color change. The color development was then stopped with the addition of 100 *μ*L of stop solution. The absorbance was measured at 450 nm using a spectrophotometer (Thermo Fisher Scientific Inc. Waltham, MA, USA). 

### 2.6. Statistical Analysis

Values of outcome data are expressed as mean ± standard error of mean (SEM). Independent *t*-tests were used to examine baseline differences in body weight, uterine weight, estradiol, ACTH, and corticosterone levels between the two randomized groups (OVX and sham-operated control). Effects of social stress (stressed versus non-stressed) and surgery (sham-operated versus OVX) on behaviour were analyzed using two-way analyses of variance (ANOVA). Pearson's correlation coefficient was utilized to test the correlation between the estradiol, ACTH, and corticosterone levels and memory, anxiety-like, and depressive-like behavioural scores. Differences were taken to be significant at *P* < 0.05. 

## 3. Results

### 3.1. Body and Uterine Weights

The weight gain was significantly higher in OVX than that in sham-operated control groups. The uterine weight after OVX was significantly lower than that in sham-operated controls (Table 1).

### 3.2. Serum Estradiol, ACTH, and Corticosterone Levels

The serum estradiol levels were significantly higher in sham-operated controls than that in OVX groups (Table 2). The serum ACTH and corticosterone levels were significantly higher in stress compared to non-stress groups (Table 2). 

### 3.3. Novel Object Recognition (NOR) Test

There was significant surgery effect on discriminative index during STM (*F*(1,36) = 11.21; *P* = 0.002) and LTM (*F*(1,36) = 62.98; *P* = 0.000) tests, indicating that OVX was associated with both short-term and long-term memory deficits. However, a significant stress effect was only noted on discriminative index during LTM (*F*(1,36) = 34.65; *P* = 0.000) but not during STM (*F*(1,36) = 3.75; *P* = 0.061) tests, indicating that stress was associated with long-term memory deficit.

**Figure 1:**
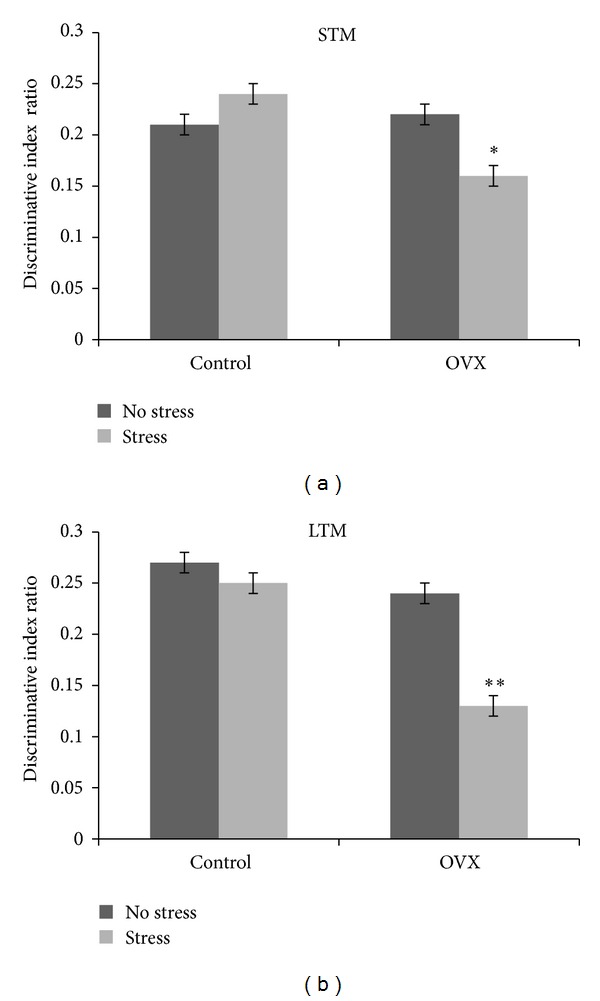
Effect of social instability stress on discriminative index ratio of short-term memory (STM) and long-term memory (LTM) tests in sham-operated control (control) and ovariectomised (OVX) rats. Each column represents the mean ± SEM of 10 rats; significantly different from non-stressed group at *P < 0.05; **P < 0.001. Control: sham-operated control rats; OVX: ovariectomised rats.

With regard to interaction effect between stress and OVX, there were significant interactions in both STM (*F*(1,36) = 24.85; *P* = 0.000) and LTM (*F*(1,36) = 16.88; *P* = 0.000) tests, indicating that sham-operated control and OVX rats were affected differently by stress. Specifically, significant short-term and long-term memory deficits were observed in OVX rats but not in sham-operated controls following stress (Figure 1).

### 3.4. Open Field Test (OFT)

Analysis by two-way ANOVA showed a significant stress effect on the all anxiety-like behaviour such as on the total rearing number (*F*(1, 36) = 11.38, *P* = 0.002), grooming (*F*(1, 36) = 50.30, *P* = 0.000), time spent crossing the lines (*F*(1, 36) = 16.93, *P* = 0.001), freezing time (*F*(1, 36) = 25.79, *P* = 0.000), and number of boli (*F*(1, 36) = 9.90, *P* = 0.003), except for face washing behaviour (*F*(1, 36) = 1.31, *P* = 0.259), indicating that stress was associated with higher anxiety-like behaviour. 

Our data also showed a significant surgery effect on all anxiety-like behaviour such as total rearing number (*F*(1, 36) = 68.26, *P* = 0.000), grooming (*F*(1, 36) = 39.76, *P* = 0.000), time spent crossing the lines (*F*(1, 36) = 226.26, *P* = 0.000), freezing time (*F*(1, 36) = 314.42, *P* = 0.000) and number of boli (*F*(1, 36) = 75.08, *P* = 0.000), except for face washing behaviour (*F*(1, 36) = 3.83, *P* = 0.058), indicating that OVX was also associated with higher anxiety-like behaviour.

**Figure 2:**
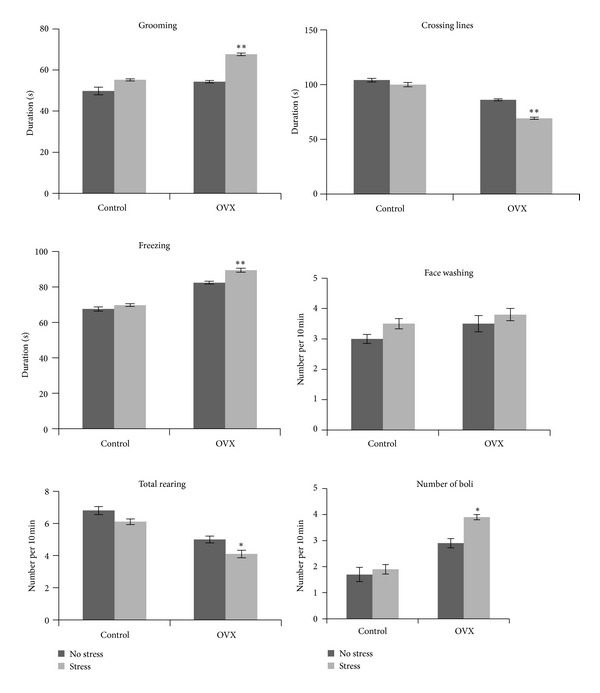
Effect of social instability stress on anxiety behaviour in sham-operated control (control) and ovariectomised (OVX) rats. Each column represents the mean ± SEM of 10 rats; significantly different from non-stressed group at *P < 0.05; **P < 0.001. Control: sham-operated control rats; OVX: ovariectomised rats.

Except for face washing behaviour (*F*(1, 36) = 1.67, *P* = 0.204), there were significant interactions between stress and OVX in most of the anxiety-like behaviour such as on total rearing number (*F*(1, 36) = 4.06, *P* = 0.049), grooming (*F*(1, 36) = 7.55, *P* = 0.009), time spent crossing the lines (*F*(1, 36) = 9.38, *P* = 0.004), freezing time (*F*(1, 36) = 7.69, *P* = 0.009), and number of boli (*F*(1, 36) = 4.13, *P* = 0.048), indicating that sham-operated control and OVX rats were affected differently by stress. Most of the anxiety-like behaviour was significantly increased in OVX rats but not in sham-operated controls following stress (Figure 2). 

### 3.5. Forced Swim Test (FST)

Our data revealed a significant surgery effect on the total swimming time (*F*(1, 36) = 57.82, *P* = 0.000), immobility time (*F*(1, 36) = 358.84, *P* = 0.000), and struggling time (*F*(1, 36) = 694.24, *P* = 0.000), indicating that OVX was associated with more depressive-like behaviour. However, a significant stress effect was observed on immobility time (*F*(1, 36) = 4.45, *P* = 0.046) and struggling duration (*F*(1, 36) = 4.21, *P* = 0.047) but not on swimming time (*F*(1, 36) = 4.03, *P* = 0.052), indicating that stress was associated with more depressive-like behaviour.

**Figure 3:**
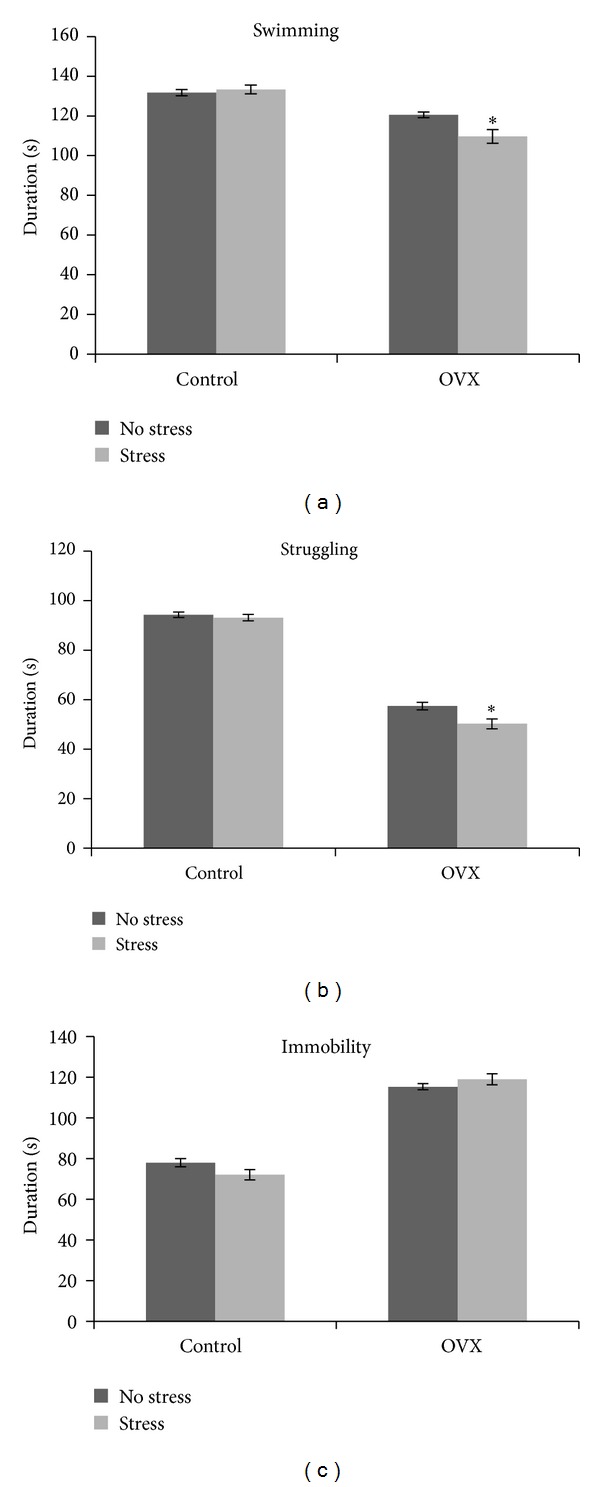
Effect of social instability stress on depressive behaviour in sham-operated control (control) and ovariectomised (OVX) rats. Each column represents the mean ± SEM of 10 rats; significantly different from non-stressed group at *P < 0.05; Control: sham-operated control rats; OVX: ovariectomised rats.

There were significant interactions between stress and OVX in all the depressive-like behaviour such as immobility time (*F*(1, 36) = 4.57, *P* = 0.039), struggling time (*F*(1, 36) = 5.36, *P* = 0.026), and swimming time (*F*(1, 36) = 7.34, *P* = 0.011), indicating that sham-operated control and OVX rats were affected differently by stress. The depressive-like behaviour were significantly increased following stress in OVX rats but not in sham-operated controls (Figure 3).

### 3.6. Correlation between the Estradiol, ACTH, and Corticosterone Levels and the Memory, Anxiety-Like, and Depressive-Like Behavioural Scores

There were significant correlations between the estradiol, ACTH, and corticosterone levels, and most of the memory, anxiety-like, and depressive-like behavioural scores except for STM and face washing anxiety-like behaviour (Table 3). There were no significant correlations between the estradiol levels and STM, and between the estradiol levels and face washing behavioural scores. Additionally, there was no significant correlation between the corticosterone levels and STM scores.

## 4. Discussion

The present study used ovariectomy and stress to investigate the emotional and cognitive behavioural changes underlying the interactional effects of estrogen and HPA axis. The OVX rats in the present study had significantly lower estradiol level compared to those of sham-operated controls. Furthermore, our studies showed that the weight gain in OVX rats was significantly higher than that in sham-operated controls and the uterine weight in OVX rats was significantly lower than that in sham-operated controls. OVX rats demonstrated body weight gain and a significant loss in uterine weight, which has previously been used as a reliable measure of estrogen deprivation at the end-organ sites [60, 61].

The present study showed that the overall memory deficit and anxiety- and depressive-like behaviour were increased in OVX than sham-operated control rats, indicating that deficiency in ovarian hormones enhanced mood and memory dysfunction. These observations were in line with previous findings that showed removal of endogenous estrogen from female rats produced memory deficit and anxiety- [29, 62] and depressive-like behaviour, and these effects can be prevented by hormone replacement [62, 63].

In addition, study conducted by Okada et al. [64] demonstrated less depressive-like behaviour in sham-operated than that in OVX rats as seen in our study. They also found that changes in OVX rats were ameliorated by estradiol (E2) administration, suggesting that E2 displayed antidepressive action only when it is in the physiological range and E2 shares some biochemical properties that typical antidepressants possess, for example, β-receptor down regulation [65] and inhibition of monoamine oxidase activity [66].

Our biochemical data confirmed that serum ACTH and corticosterone levels in animals exposed to social instability stress for 15 days were significantly higher than that in non-stressed rats [67]. Moreover, the overall anxiety- and depressive-like behaviour were increased in stressed compared to non-stressed rats. This could be due to alteration in the interaction between hypothalamic-pituitary-adrenal (HPA) axis and the serotonergic (5-HTergic) systems following chronic stress. The dysfunction of both systems may partly explain the neurobiology of emotional dysfunction in animals that have been exposed to stress [68].

The memory deficit was also increased in stressed compared to non-stressed rats. Studies have shown that the prefrontal cortex and limbic system play a modulatory role in the response of HPA and sympathetic-adrenal-medullary axes to psychological stressors [69, 70]. The hippocampus, which participates in cognitive processes, is also connected to the HPA axis and seems to be particularly vulnerable to stress [70–72]. In fact, with prolonged chronic stress, the HPA axis is hyperactivated, with the resulting release in ACTH and cortisol/corticosterone, thus involving structural changes, cell atrophy and neuronal loss in the hippocampus [70, 73, 74]. Glucocorticoid effects on memory consolidation follow an inverted U-shape dose-response relationship; moderate doses enhance memory, whereas higher doses are typically less effective or may even impair memory consolidation [75] as seen by impaired long-term memory in our chronically stressed rats.

Interestingly, memory deficit and anxiety- and depressive-like behaviour were increased in stressed OVX compared to the other groups. These findings suggest that social stress and surgery which is a form of physical stress are dependent factors as supported by the significant interactions and correlations between OVX and stress in most of the rats' behaviours. As in the previous studies, ovarian hormones are known to regulate the plasma levels of ACTH and corticosterone, the basal and stress-induced activity of the HPA axis [76], and the expression of mineralocorticoid receptors in the hippocampus [77], suggesting that ovarian hormones influence neural tissue respond to stress. 

Our findings, however, were in contrast to the findings reported by Zhang and colleagues [78]. They reported that anxiety-like behaviour was increased with psychological stress only in the sham-operated rats but not in the OVX rats. They concluded that female gonadal hormones may play an important role in the regulation of brain 5-HTergic systems. These interactions between gonadal hormones and 5-HT metabolism may play a role in 5-HT-related neuropsychiatric disorders. The discrepancies could be explained by the difference in duration following OVX and duration and type of psychological stress. In the present study, behavioural tests were performed at 8 weeks compared to 2 and 4 weeks following OVX in the previous study. The psychological stress used in the previous study was foot shocks for 1 hour every day for 5 days as compared to our study whereby chronic instability in the housing conditions by alternating isolation and crowding phases for 15 days was utilized. However, there was no data available on the estradiol and corticosterone levels in that study.

## 5. Conclusions

Memory deficit and anxiety- and depressive-like behaviour were significantly increased in stressed OVX compared to non-stressed OVX rats but the same changes were not seen in sham-operated control rats. It is possible that the high circulating corticosterone acts synergistically with low circulating estradiol to exert negative effects on mood and memory function. These findings support the significance of additional factor such as social stress in the development of postmenopausal memory deficits as well as anxiety and depressive symptoms.

## Figures and Tables

**Table 1:** Body weight, uterine weight and, serum estradiol level in sham-operated control and ovariectomised groups.

Group	Body weight gain (g)	Uterine weight (g)	Estradiol (pg/mL)
Control	24.200 ± 0.247	0.694 ± 0.007	230.100 ± 11.296
OVX	59.600 ± 2.716**	0.590 ± 0.007**	81.800 ± 2.913**

Each value represents the mean ± SEM of 10 rats; significantly different from control at *P < 0.05; **P < 0.001. Control: sham-operated control rats; OVX: ovariectomised rats.

**Table 2:** Serum ACTH and corticosterone levels in non-stressed and stressed groups.

Group	ACTH (pg/mL)	Corticosterone (pg/mL)
Non-stressed	45.789 ± 1.200	4611.000 ± 169.430
Stressed	56.952 ± 1.891**	5661.905 ± 163.951**

Each value represents the mean ± SEM of 10 rats; significantly different from non-stressed group at *P < 0.05; **P < 0.001. Control: sham-operated control rats; OVX: ovariectomised rats.

**Table 3:** Correlation between the estradiol, ACTH, and corticosterone levels and the memory, anxiety- and depressive-like behavioural scores.

	Estradiol (pg/mL)	ACTH (pg/mL)	Corticosterone (pg/mL)
Memory			
Discriminative index ratio of STM	0.202	−0.435*	−0.222
Discriminative index ratio of LTM	0.444*	−0.697**	−0.715**
Anxiety-like behaviour			
Grooming (s)	−0.584**	0.758**	0.792**
Cross lines (s)	−0.774**	0.683**	0.812**
Freezing (s)	−0.783**	0.673**	0.834**
Face washing (number per 10 min)	−0.287	0.363*	0.442*
Total rearing (number per 10 min)	0.734**	−0.597**	−0.743**
No of boli (number per 10 min)	−0.734**	0.671**	0.767**
Depressive-like behaviour			
Swimming (s)	0.722**	−0.540**	−0.720**
Struggling (s)	0.858**	−0.428*	−0.745**
Immobility (s)	−0.821**	0.468*	0.728**

Each value represents the Pearson correlation coefficients (*r*). *P < 0.05; **P < 0.001.
